# Strategy investments in zero-sum games

**DOI:** 10.1007/s11590-024-02130-z

**Published:** 2024-06-24

**Authors:** Raul Garcia, Seyedmohammadhossein Hosseinian, Mallesh Pai, Andrew J. Schaefer

**Affiliations:** 1 Department of Computational Applied Mathematics and Operations Research, Rice University, Houston, TX 77005 USA; 2 Department of Mechanical and Materials Engineering, University of Cincinnati, Cincinnati, OH 45221 USA; 3 Department of Economics, Rice University, Houston, TX 77005 USA

**Keywords:** Zero-sum games, Matrix games, Strategy investments, Mixed-integer linear programming

## Abstract

We propose an extension of two-player zero-sum games, where one player may select available actions for themselves and the opponent, subject to a budget constraint. We present a mixed-integer linear programming (MILP) formulation for the problem, provide analytical results regarding its solution, and discuss applications in the security and advertising domains. Our computational experiments demonstrate that heuristic approaches, on average, yield suboptimal solutions with at least a 20% relative gap with those obtained by the MILP formulation.

## Introduction

We consider a two-player zero-sum game, where each player selects an action from a finite set of available actions. The payoffs to each player depend on the actions selected by both. The game is zero-sum, as the amount won by one player is the loss of the other, i.e., the payoffs always sum to zero. The payoffs in such a game can be captured by a matrix whose rows and columns identify the actions available to the players and entries specify the amount of winnings/losses; hence, the game is also called a *matrix game*. The matrix is fixed and known to the players. It is customary to present the matrix by the earnings of the row player; a negative entry indicates a loss.

Given a matrix $A = [a_{ij}]_{m \times n}$ representing the game, the row and column players simultaneously select *strategies* to maximize their payoff from the game, accounting for strategic play by the other player. Let $x \in \mathbb {R}^{m}_{+}$ denote the row player’s strategy, i.e., they select action $i \in \{1,\ldots ,m\}$ with probability $x_i$, where $\sum _{i=1}^m x_i = 1$. Similarly, let $y \in \mathbb {R}^{n}_{+}$ denote the column player’s strategy, unknown to the row player. When a player’s strategy is a unit vector, it is called a *pure* strategy; otherwise, it is called a *mixed* strategy. The expected payoff to the row player in each round is $x^\top A y = \sum _{i=1}^m \sum _{j=1}^n a_{ij} x_i y_j$. Patently, with a fixed strategy $\hat{x}$, the row player’s expected earning will be no less than $\min \limits _{y} \hat{x}^\top A y$, which is shown to be equal to $\min \limits _{j} \sum _{i=1}^m a_{ij} \hat{x}_i$ [1]. Thus, an optimal strategy for the row player can be obtained by maximizing this lower bound on their expected earning over all possible strategies, as follows:

1 $$\begin{aligned} \begin{aligned} \max \limits _{x,z}&\quad z \\ \text {s.t.}&\quad z - \sum _{i=1}^m a_{ij} x_i \le 0, \quad \forall j \in \{1,\ldots ,n\},\\&\quad \sum _{i=1}^m x_i = 1, \\&\quad x_i \ge 0, \quad \forall i \in \{1,\ldots ,m\}. \end{aligned} \end{aligned}$$

The optimal value of linear program (1) is called the *value* of the game. By duality, it can be shown that an optimal strategy for the column player that minimizes their maximum expected loss leads to the same game value; this is the celebrated Minimax Theorem [2]. We refer to [1, 3] for more details on matrix games.

In this work, we consider a novel extension of matrix games. The row player is given a budget and must pay for each row they wish to have available and for each column they may wish to remove. That is, there is a given payoff matrix *A*, where each row and column have an associated price. The row player first selects a (budget-feasible) submatrix of *A*, and the game is then played on the submatrix. Hence, the row player seeks to determine an optimal set of row selections and column removals to purchase and a corresponding (mixed) strategy for the game. We call this problem the *matrix game designer problem* (MGD) and show it can be formulated as a mixed-integer linear program (MILP).

We provide analytical results concerning the formulation, as well as the results of our computational experiments. Our analytical results highlight that MGD is substantially different than the problem of finding optimal strategies in a fixed zero-sum game. For example, it is well known that in zero-sum games, dominated actions are never played in an optimal strategy.^1^ Nevertheless, we show that in MGD, depending on the associated costs, dominated strategies may be purchased and played, while the corresponding dominating strategy is not. Our computational results then show that our MILP formulation lends itself to “good” solutions using an off-the-shelf MILP solver, i.e., ones that can be computed reliably in reasonable time on randomly drawn games. In contrast, we show that multiple natural heuristic approaches that one may apply to MGD are substantially suboptimal relative to the solutions calculated via the MILP approach.

Our work is primarily motivated by applications of MGD in the security domain and is similar to existing Bayesian Stackelberg game approaches to security scheduling [4–7]. In these settings, a limited set of security resources must be scheduled to maximize some notion of rewards, which are generally constructed to capture the severity of different types of attacks and may incorporate their probability of success. A Stackelberg game is a general-sum matrix game where a leader (e.g., defender) moves first and a follower (e.g., attacker), possibly having learned the leader’s strategy, moves after. Bayesian Stackelberg games extend this to account for multiple follower types, the realized type unknown to the leader beforehand. While Stackelberg games have successfully been employed in the scheduling of canine units at US airports [4], US Air Marshalls on commercial flights [8], and patrol units to prevent wild life poaching [9], current approaches assume a fixed set of resources which may be readily selected with no explicit associated expenses.

We differ from previous works in security games in that we do not assume possession of a fixed set of resources readily available for use, but instead consider a budgetary constraint reflecting investments into specific types of security. That is, whereas previous works focus solely on scheduling existing resources, we instead model the problem of determining which resources to invest into as well as how to employ them. Another important difference is our use of zero-sum games, which, although less general, exhibit favorable properties in their resolution by admitting a MILP formulation without requiring ancillary linearization techniques. Further, we do not explicitly consider multiple attacker types, but instead allow for arbitrary types of attacks. Additionally, we show that our strategy investment framework can be extended to the Bayesian Stackelberg security game formulation of Pita et al. [4].

## Applications

We provide two applications for our framework within the security and advertising domains.

First, consider securing an airport against malicious, strategic attackers. There is a set of *m* available screening technologies (e.g., millimeter wave scanners) or security personnel, which can be employed at particular locations [10, 11]. There is also a set of *n* possible attacks against the airport. Screening technology *i* interdicts attack *j* with a given probability $p_{ij}$. These probabilities constitute a matrix *A*. We assume that the airport wants to maximize the probability of successful interdiction while the attackers wants to minimize the same objective, meaning that the matrix *A* can be interpreted as the payoff matrix of a zero-sum game. This framework can be easily extended to account for severity of the attacks by defining $a_{ij} = R_j (p_{ij})$, where $R_j$ is a weight parameter indicating the importance of preventing attack *j*. We can extend the above setting to also accommodate various operational conditions. For example, if the effectiveness of certain security measures vary under different operational conditions (e.g., less likelihood of detecting malicious events during rush hours), an attack can be considered as two separate actions from the opponent (during rush hours and otherwise), with different conditional probabilities for an effective detection.

In addition to the screening technologies varying in price and effectiveness (e.g., basic x-ray machines versus new 3D computational tomography scanners), there may also be available investments which (at a cost) serve to remove certain feasible actions of the attackers. For instance, investment in high compound walls or vehicle security barriers may remove the possibility of certain perimeter breaches [12]. With a given budget, the airport must first choose which security measures to make available and/or which actions of the attackers to eliminate. Subsequently the airport must choose how to employ the chosen screening techniques (optimal strategy) to maximize the likelihood of detecting malicious attacks, taking into account that attackers will be strategic. Given a set of available screening technologies, a strategy *x* corresponds to the frequency of assignments of passengers to the different types of security technologies and procedures.^2^ Any given attacker then chooses an attack *j*, so that the proportion of various attacks in the population is denoted *y*.

As a second application, we consider a model of competition in online advertising. Consider an entity, such as a candidate running for election or a company selling a specific product, seeking an advertising strategy on an online platform. Every user of the platform receives an advertisement from the entity and an advertisement from its opponent. Depending on the pair of advertisements the user sees, the user will align with the entity (i.e., vote for the candidate or purchase the entity’s product) with some probability and with the competitor with complementary probability. Similar to the previous application, these probabilities form a matrix *A*, which defines a zero-sum game between the entity and its opponent. The entity’s objective is to maximize the probability of the user aligning with them. In line with the motivation of our paper, these advertising options are not directly available to the entity— instead, each has an associated investment cost. For instance, the cost could be the production cost of a 30-second video, which depends on factors such as the choice of celebrities, production team, locations, etc. Therefore, with a fixed budget, they need to decide which advertisement options to produce and how frequently to use them (optimal strategy) to maximize the likelihood of their success.

## Formulation

Consider a matrix game *A* and the respective formulation (1). We extend this game by introducing variables $r \in \{0,1\}^m$ and $s \in \{0,1\}^n$ to specify the designer’s decisions; $r_i = 1$ indicates row $i \in \{1,\dots ,m\}$ has been selected, and $s_j = 1$ indicates column $j \in \{1,\ldots ,n\}$ has been removed. Let $c^r \in \mathbb {R}^m_{+}$ and $c^s \in \mathbb {R}^n_{+}$ denote the (nonnegative) cost vectors associated with purchasing rows and eliminating columns, respectively, and *b* the resource budget. The following proposition captures the representability of MGD as a mixed-integer linear program (MILP).

### Proposition 1

Given a matrix game $A = [a_{ij}]_{m \times n}$ and a budget *b*, the corresponding matrix game designer’s problem (MGD) can be formulated as follows:

2a $$\begin{aligned} \max \limits _{r,s,x,z}&\quad z \end{aligned}$$

2b $$\begin{aligned} \text { s.t.}&\quad z - \sum _{i=1}^m a_{ij} x_i \le M_j s_j, \quad \forall j \in \{1,\ldots ,n\}, \end{aligned}$$

2c $$\begin{aligned}&\quad \sum _{i=1}^m x_i = 1, \end{aligned}$$

2d $$\begin{aligned}&\quad \sum _{i=1}^{m} c^{r}_{i} r_{i} + \sum _{j=1}^{n} c^{s}_{j} s_{j} \le b, \end{aligned}$$

2e $$\begin{aligned}&\quad 0 \le x_i \le r_{i}, \quad \forall i \in \{1,\ldots ,m\}, \end{aligned}$$

2f $$\begin{aligned}&\quad r \in \{0,1\}^{m}, \end{aligned}$$

2g $$\begin{aligned}&\quad s \in \{0,1\}^{n} , \end{aligned}$$

where $M_j = a_{\max } - \min \limits _{i} a_{ij},~\forall j \in \{1,\ldots ,n\}$, and $a_{\max }$ denotes the largest entry of matrix *A*.

The formulation is equivalent to (1), with additional constraints: (2d) together with (2f)-(2g) enforces the budget constraint, and (2e)-(2f) ensure the row player may only play rows purchased. The parameter $M_j$ in (2b) ensures that the corresponding constraint associated with an eliminated column is relaxed. To show that $M_j=a_{\max } - \min \limits _{i} a_{ij}$ is an upper bound on $z - \sum _{i=1}^m a_{ij} x_i$, $\forall j \in \{1,\ldots ,n\}$, observe that the value of a game is always bounded above by its largest matrix entry, i.e., $z \le a_{\max }$, and for every $j \in \{1,\ldots ,n\}$, $\sum _{i=1}^m a_{ij} x_i \ge (\min \limits _{i} a_{ij}) \sum _{i=1}^{m} x_i = \min \limits _{i} a_{ij}$.

### Remark 1

We naturally assume that the budget *b* can afford to purchase each row separately, i.e., $b \ge \max \{c^{r}_{i}: i=1,\ldots ,m\}$; otherwise, rows whose prices exceed *b* may be removed from the formulation. We also assume the budget is not sufficient to eliminate all columns of the original game, i.e., $b < \sum _{j=1}^{n} c^{s}_{j}$, because otherwise the problem is trivial (i.e., remove all columns). Further, note that constraint (2c) combined with (2e) ensures that at least one row is purchased. Under these conditions, it can be easily verified that formulation (2) is always feasible and well-defined.

We highlight that the values of $M_j, \, j \in \{1,\ldots ,n\}$, play a crucial role in the strength of an MILP formulation. Larger values increase the size of the convex relaxation, which generally results in a larger search tree [13, 14]. Therefore, it is desirable to obtain values for $M_j$ which are as tight as possible.

### Remark 2

$M_j$ is a sharp bound on $z - \sum _{i=1}^m a_{ij} x_i,~\forall j \in \{1,\ldots ,n\}$. Note that an optimal solution to (2) may constitute a matrix of a single row and a single column. Let $i' \in \{1,\ldots ,m\}$ and $j' \in \{1,\ldots ,n\}$ respectively identify the actions available to the row and column players in such a game, with $a_{i'j'} = a_{\max }$, which implies $x_{i'}^* = 1$ and $z^* = a_{\max }$. Then, for a column $j'' \in \{1,\ldots ,n\} \backslash \{j'\}$, the condition $a_{i'j''} = \min \limits _{i} a_{ij''}$ will suffice to lead to $z^* - \sum _{i=1}^m a_{ij''} x_i^* = a_{\max } - \min \limits _{i} a_{ij''}$. Any further improvement on this bound will depend on additional information, such as practicality and affordability of removing particular columns. The impracticality of removing a column *j* may be enforced by setting $M_j = 0$ or enforcing $s_j = 0$.

## Analytical results

Our first result concerns the standard game-theoretic notion of *dominated* actions. An action *i* is said to be *dominated* (by action $i'$) if there exists another action $i'$ for the player which gives them a higher payoff regardless of the opponent’s action. In regular matrix games, dominated actions may be discarded from the formulation, as an optimal strategy will never employ them. In MGD, however, the purchasing price must also be taken into account.

### Proposition 2

Let a row *i* of a matrix game *A* be dominated by some row $i'$. Then, an optimal solution to an instance of MGD characterized by matrix *A* and budget *b* (I) will never include both *i* and $i'$, and (II) may include *i* only if it is strictly less expensive to purchase than $i'$, i.e., $c^r_i < c^r_{i'}$.

### Proof

The proof of (I) is implied by the dominance of $i'$ in the game over a submatrix including both *i* and $i'$. For (II), let row *i* be at least as expensive to purchase as row $i'$ and included in an optimal solution of MGD with (optimal) strategy $\tilde{x}$, i.e., $\tilde{x}_i > 0$ and $\tilde{x}_{i'} = 0$. Then, replacing *i* with $i'$ in such a solution will yield a budget-feasible strategy $\hat{x}$ with $\hat{x}_i = \tilde{x}_{i'} = 0$, $\hat{x}_{i'} = \tilde{x}_i > 0$ and a larger game value, contradicting optimality of $\tilde{x}$.

An immediate implication of this result is that a dominated action for the row player in the original matrix game may be excluded from the MGD formulation only if it is at least as expensive to purchase as the corresponding dominating action.

### Remark 3

It is rational to assume that the opponent (column player) will never select a dominated action from their set of available actions (i.e., a column whose every entry yields a higher payoff to the row player than some other column). However, since the set of available actions for the column player is determined by the row player in MGD, a dominated column (of the original game) may not be discarded from the formulation; such a column may prove to be a part of the column player’s optimal strategy in the absence of the dominating column, due to its removal.

The concept of row dominance in MGD may be extended to tighten the linear-programming relaxation solution of (2), as shown by the following proposition.

### Proposition 3

Let *A* be a matrix game such that row *i* contains equal or lesser payoffs (to the row player) than a row $i'$, for all of the opponent’s actions except for a column $j'$, i.e., $a_{ij} \le a_{i'j},~\forall j \in \{1,\ldots ,n\} \backslash \{j'\}$. We say row *i* is conditionally dominated by row $i'$. Then, if $c^r_i \ge c^r_{i'}$, the inequality $x_i + s_{j'} \le 1$ is valid for (2).

### Proof

The proof is almost immediate. If $s_{j'} = 0$ in an optimal solution of MGD, the inequality is trivially valid. On the other hand, if $s_{j'} = 1$ (i.e., the column $j'$ is removed), row *i* may be safely excluded from consideration by Proposition 2, leading to the valid inequality $x_i \le 0$. $\square$

We provide computational results on the strength of these inequalities in Appendix A.

With a view to defining a heuristic approach to MGD in the sequel, we can define what we term the *attractiveness* of actions. This is a more nuanced concept than dominance; the latter is the case where an action is more attractive to a player than another action, regardless of that of the opponent. In particular, upon solving a regular matrix game, we consider the actions’ *frequency of use* in an optimal strategy as a measure of their attractiveness: we say an action is more attractive than another if it is to be played more frequently in an optimal strategy of the game.^3^

Recall that frequency of use for the row and column player’s actions in a regular matrix game are given by the optimal values of the primal and dual variables of linear program (1), respectively. Such preferences, however, do not hold under the budget constraint for MGD. In fact, a row that is a part of an optimal strategy in the regular matrix game may not be selected under the budget constraint. Similarly, a column leading to a nonbinding value constraint $z - \sum _{i=1}^m a_{ij} x_i \le 0$ of the regular matrix game may become binding in an optimal solution of (2). More importantly, MGD does not exhibit a nesting behavior regarding selected rows and removed columns as the budget increases. This is illustrated through the following example.

### Example 1

Consider the matrix game

$$\begin{aligned} A = \begin{bmatrix} -0.20 &{} -0.10 &{} 0.20 &{} 0.20 &{} 0.15 \\ 0.00 &{} 0.35 &{} 0.10 &{} -0.10 &{} 0.20 \\ 0.30 &{} 0.35 &{} -0.05 &{} -0.25 &{} -0.05 \\ 0.25 &{} 0.25 &{} -0.25 &{} -0.05 &{} 0.40 \\ 0.45 &{} -0.15 &{} 0.20 &{} -0.20 &{} 0.10 \end{bmatrix} \end{aligned}$$

with row and column prices given by

$$\begin{aligned} \begin{aligned}&c^r = \begin{bmatrix} 2&3&4&4&5 \end{bmatrix},~\text {and}\\&c^s = \begin{bmatrix} 10&11&12&11&12 \end{bmatrix}, \end{aligned} \end{aligned}$$

respectively. Let *R* and *S* denote the sets of selected rows and removed columns, respectively. With a budget of 20 units, the (unique) optimal purchasing decisions and strategy are

$$\begin{aligned} R = \{2,5 \}, \quad S = \{4 \}, \\ x = \begin{bmatrix} 0.00&0.58&0.000&0.00&0.42 \end{bmatrix}, \end{aligned}$$

leading to the optimal objective value of 0.14. With an increased budget of 25 units, the (unique) optimal purchasing decisions and strategy are

$$\begin{aligned} R = \{1 \}, \quad S = \{1,2 \}, \\ x = \begin{bmatrix} 1.00&0.00&0.00&0.00&0.00 \end{bmatrix}, \end{aligned}$$

leading to the optimal objective value of 0.15. On the other hand, in the regular matrix game the row player would employ actions $\{1,2,4,5\}$ and obtain an objective of 0.04. The corresponding optimal strategy is given by

$$\begin{aligned} x = \begin{bmatrix} 0.47&0.08&0.00&0.33&0.12 \end{bmatrix}. \end{aligned}$$

This observation indicates that a solution method for purchasing decisions that follows some measure of preference inherited from the original matrix game would lead to suboptimal strategies for MGD in general. Similarly, a greedy method for row selection and column removal decisions based on immediate gain in the game value would generally be suboptimal for MGD. These approaches, however, may be used to construct heuristic solutions for the problem. We investigate the quality and computational performance of these methods in comparison with formulation (2) in Sect. 6.

﻿Formulation (2) can be modified to determine the minimum budget needed to achieve a game value at least *v*. This is particularly important in security games, where a certain minimum game value (probability of interdicting attacks) must be achieved. We note that meaningful values for *v* will depend on the original matrix game. For example, setting $v > a_{\max }$ leads to patent infeasibility.

### Proposition 4

Given a matrix game $A = [a_{ij}]_{m \times n}$, the problem of determining the minimum budget required to achieve a game value at least *v*—or conclude infeasibility—may be formulated as follows:

3a $$\begin{aligned} \min \limits _{r,s,x,\beta }&\quad \beta \end{aligned}$$

3b $$\begin{aligned} \text { s.t.}&\quad v - \sum _{i=1}^m a_{ij} x_i \le M_j s_j, \quad \forall j \in \{1,\ldots ,n\}, \end{aligned}$$

3c $$\begin{aligned}&\quad \sum _{i=1}^m x_i = 1, \end{aligned}$$

3d $$\begin{aligned}&\quad \sum _{i=1}^{m} c^r_i r_i + \sum _{j=1}^{n} c^s_j s_j \le \beta , \end{aligned}$$

3e $$\begin{aligned}&\quad 0 \le x_i \le r_{i}, \quad \forall i \in \{1,\ldots ,m\}, \end{aligned}$$

3f $$\begin{aligned}&\quad r \in \{0,1\}^{m}, \end{aligned}$$

3g $$\begin{aligned}&\quad s \in \{0,1\}^{n} , \end{aligned}$$

where $\beta$ denotes the budget.

### Proof

Given a budget $\hat{\beta }$, consider an arbitrary partial feasible solution $(\hat{r},\hat{s},\hat{x})$ to (3) and let $\hat{I}$ and $\hat{J}$ denote the corresponding sets of selected rows and columns for the game, respectively, i.e., $\hat{I} = \{i: \hat{r}_i = 1\}$ and $\hat{J} = \{j: \hat{s}_j = 0\}$. Observe that $v \le \sum _{i=1}^m a_{ij} \hat{x}_i,~\forall j \in \hat{J},$ holds by (3b), which immediately implies $v \le \min \limits _{j \in \hat{J}} \sum _{i=1}^m a_{ij} \hat{x}_i.$ Since the choice of $\hat{x}$ was arbitrary, this result holds for all (feasible) strategies within $\hat{I}$. Therefore, every feasible solution to (3) induces a matrix game whose value is at least as large as *v*, and the formulation seeks that with the minimum budget among all such feasible solutions. $\square$

### Remark 4

While an optimal solution $(r^*,s^*,x^*,\beta ^*)$ of (3) identifies the minimum budget and optimal row selection and column removal decisions, the strategy solution $x^*$ may not lead to the value $z^* = \max \limits _{x(I^*)} \left\{ \min \limits _{j \in J^*} \sum _{i=1}^m a_{ij} {x}_i \right\}$ of the game induced by $I^*$ and $J^*$. We note that game value is a (nondecreasing) piecewise constant function of the budget. This implies that, given a partial optimal solution $(r^*, s^*, \beta ^*)$, the corresponding game value may be considerably greater than *v*, while $x^*$ only needs to ensure that the expected payoff to the row player is no less than *v*. After obtaining the minimum budget and optimal row selection and column removal decisions from an optimal solution of (3), an optimal strategy for the row player in the corresponding game may be identified by solving (1) over the respective submatrix.

Finally, we note that the MGD framework can accommodate more complex cost structures. For example, in the context of advertising, one might consider banner and video as two modes of advertisement, with each mode offering multiple options in terms of quality, content, etc. Consequently, the cost of each option would comprise a platform cost (i.e., the fee charged by the online platform for displaying banner vs. video) and a production cost. While the entity incurs the platform cost only once, each option carries its own production cost. Such a fixed-cost structure can be easily represented by introducing additional (binary) variables.

## Extension to Bayesian stackelberg security games

Bayesian Stackelberg games have been employed in scheduling security resources for various applications [4, 5, 7–9]. They are attractive models due to their ability to incorporate multiple attacker types, weigh the importance of protecting different targets appropriately, and, as the defender moves first, account for the possibility of attackers learning the defender’s strategy, by generating mixed strategies [4, 15]. We now show how to extend our strategy investment framework to the Bayesian Stackelberg game of Pita et al. [4]. They formulate the problem first as a mixed-integer bilinear program (MIBLP), which is then linearized. Given the nature of the formulation, we restrict our focus to the purchasing of row player actions (i.e., no column removals).

We first provide associated notation. In this Stackelberg security game, there is one defender and |*L*| types of attackers. The vector *x* denotes the defender’s mixed strategy and $q^l$ is the binary strategy vector of an attacker of type $l \in L$. The index set of the defender’s and attacker’s actions are denoted by *X* and $Q^l$, respectively, and their payoffs are captured in matrices $R^l$ and $C^l$, respectively. Furthermore, $p^l$ denotes the probability the defender faces an attacker of type *l*, variable $a^l$ captures the reward of an attacker of type *l*, and *M* is a sufficiently large positive scalar for relaxing constraints. The objective is to maximize the expected reward of the defender.

The initial MIBLP formulation of Pita et al. [4] is linearized through the change of variables $z^l_{ij} = x_i q^l_j$; effectively, $z^l_{ij}$ captures the defender’s probability of employing action *i* in anticipation of an attack $j \in Q^l$ from an attacker of type *l*. To impose a budget constraint with budget *b* and row prices *c*, we introduce variables $r_i \in \{0,1\}, \ \forall i \in X$, and add constraints (4h)-(4j) to their linearized reformulation, resulting in the following MILP:

4a $$\begin{aligned} \max \limits _{x,q,a}&\quad \sum _{i \in X} \sum _{l \in L} \sum _{j \in Q^l} p^l R^l_{ij} z^l_{ij} \end{aligned}$$

4b $$\begin{aligned} \text {s.t.}&\quad \sum _{i \in X} \sum _{j \in Q^l} z^l_{ij} = 1, \quad \forall l \in L, \end{aligned}$$

4c $$\begin{aligned}&\quad \sum _{j \in Q^l} z^l_{ij} \le 1, \quad \forall l \in L, \ i \in X, \end{aligned}$$

4d $$\begin{aligned}&\quad q^l_j \le \sum _{i \in X} z^l_{ij} \le 1, \quad \forall j \in Q^l, \ l \in L, \end{aligned}$$

4e $$\begin{aligned}&\quad \sum _{j \in Q^l} q^l_j = 1, \quad \forall l \in L, \end{aligned}$$

4f $$\begin{aligned}&\quad 0 \le ( a^l - \sum _{i \in X} C^l_{ij} (\sum _{h \in Q^l} z^l_{ih}) ) \le (1 - q^l_j) M, \nonumber \\&\quad \hspace{35mm} \forall j \in Q^l, \ l \in L, \end{aligned}$$

4g $$\begin{aligned}&\quad \sum _{j \in Q^l} z^l_{ij} = \sum _{j \in Q^l} z^{1}_{ij}, \quad \forall l \in L, \ i \in X, \end{aligned}$$

4h $$\begin{aligned}&\quad \sum _{i \in X} c_i r_i \le b, \end{aligned}$$

4i $$\begin{aligned}&\quad \sum _{j \in Q^l} z^l_{ij} \le r_i, \quad \forall l \in L, \ i \in X, \end{aligned}$$

4j $$\begin{aligned}&\quad r_i \in \{0, 1\}, \quad \forall i \in X, \end{aligned}$$

4k $$\begin{aligned}&\quad 0 \le z^l_{ij} \le 1 \quad \forall j \in Q^l, \ l \in L, \ i \in X, \end{aligned}$$

4l $$\begin{aligned}&\quad q^l_j \in \{0,1\}, \quad \forall j \in Q^l, \ l \in L, \end{aligned}$$

4m $$\begin{aligned}&\quad a^l \in \mathbb {R}, \quad \forall l \in L. \end{aligned}$$

Note that this formulation computes a strategy $z^l_{ij}$ for each attacker type *l*, partitioned over attacker actions $j \in Q^l$; accordingly, $\sum _{j \in Q^l} z^l_{ij}$ represents the defender’s mixed strategy component $x_i$, against an attacker of type *l*. The constraint (4g) ensures the defender’s strategy is consistent across all attacker types. In (4f), the left inequality implies $a^l$ is an upper bound on the attacker’s reward, $\sum _{i \in X} C^l_{ij} (\sum _{h \in Q^l} z^l_{ih})$. The right inequality is relaxed when $q^l_j = 0$ and lower bounds the attacker’s reward with $a^l$ when $q^l_j = 1$; this results in $a^l$ necessarily equaling the attacker’s maximum payoff. The constraint (4d) ensures the defender protects against the best attacker strategy. The constraints (4h)-(4j) impose availability and budget constraints on the defender’s mixed strategy.

## Computational experiments

Finally, we describe the procedures and results of our computational experiments. In particular, we analyze the role of matrix size, budget, and column prices in the MGD formulation (2), and compare its performance with two heuristic approaches.

We constructed matrices of sizes given by the Cartesian product $\{10,50,100\}^2$. The entries were drawn uniformly from $-0.5$ to 0.5, and each column was scaled by a random number from the set $\{10, 20, 30, 40, 50\}$ to mimic severity of attacks, as explained in Sect. 2. For each matrix size, we constructed five instances. To observe the effect of row and column prices, we solved each matrix instance with row and column prices of comparable size, as well as with column prices three times and five times as large. We will refer to these column price levels as LOW, MEDIUM, and HIGH. Finally, for each matrix instance and price range, we solved the MGD with a budget 0.25, 0.50 and 0.75 the total sum of row and column prices, resulting in 405 total instances. All were generated with Julia 1.8 and solved with Gurobi 10.0.0 [16] on a machine with an Intel Core i7 processor at 1.8 GHz and 8 GB of RAM. A time limit of 20 min and a MIPGap of 1% were imposed.

**Fig. 1:**
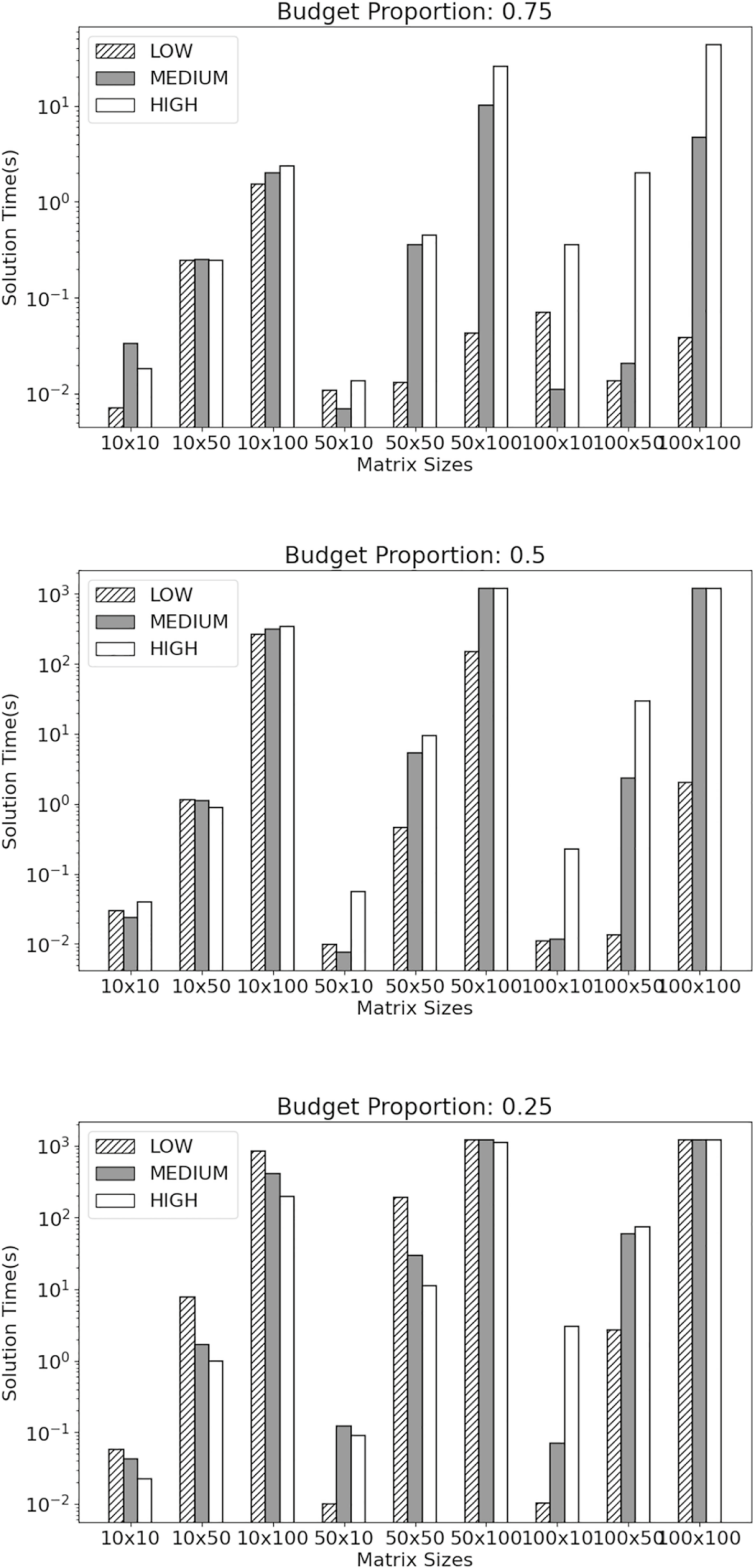
Solution time vs matrix size per column price level, for a fixed budget proportion

Figure 1 summarizes the performance of the MILP formulation (2) on the aforementioned MDG instances. This figure shows the average solution time for each class of instances. In general, the solution time exhibited a decreasing trend with increasing available budget. This was more notable when the budget proportion increased from 0.50 to 0.75. Also, higher column prices led to longer solution times, although this was less often the case for the smallest budget proportion. We note that these figures include data from instances that terminated due to the time limit (approximately 13%), resulting in smaller averages for the more difficult problems.

Next, we describe our heuristic methods. The first method is a greedy algorithm, in which row selections and column removals are purchased incrementally based on the largest immediate gain in value, as outlined Algorithm 1, where *R* denotes the set of row selections and *S* the set of column removals.

**Algorithm 1:**
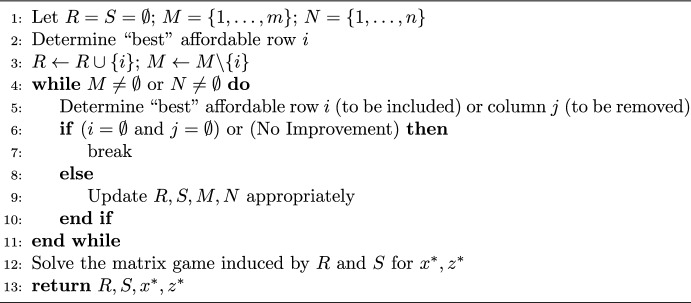
A greedy heuristic method for MGD

**Algorithm 2:**
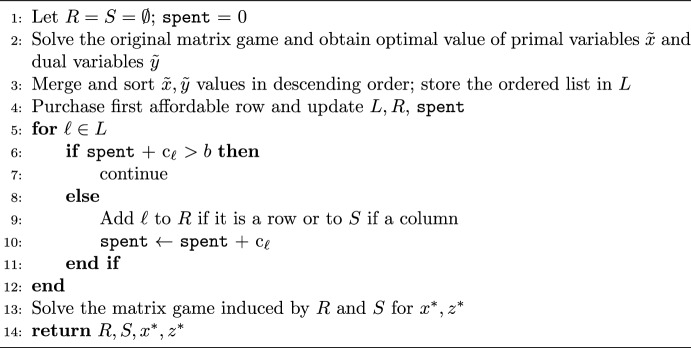
A rank-based heuristic method for MGD

In this algorithm, the “best” row selection or column removal decisions are those leading to the highest added value to an incumbent game, which may be determined by solving an MILP or a series of LPs for each unpurchased row and column. We note that purchasing at least one row is required to ensure the feasibility of the obtained solution; hence, the algorithm starts by purchasing a row. We will refer to this method as $H_\text {G}$.

Our second heuristic method exploits optimal solutions to the original matrix game (without budget); it ranks the players’ actions (rows and columns) based on their “frequency of use” as given by an optimal solution, as a measure of their attractiveness to the respective players, and follows this rank to make purchasing decisions as long as the budget allows. Algorithm 2 presents this method, which we will refer to as $H_\text {R}$.

**Fig. 2:**
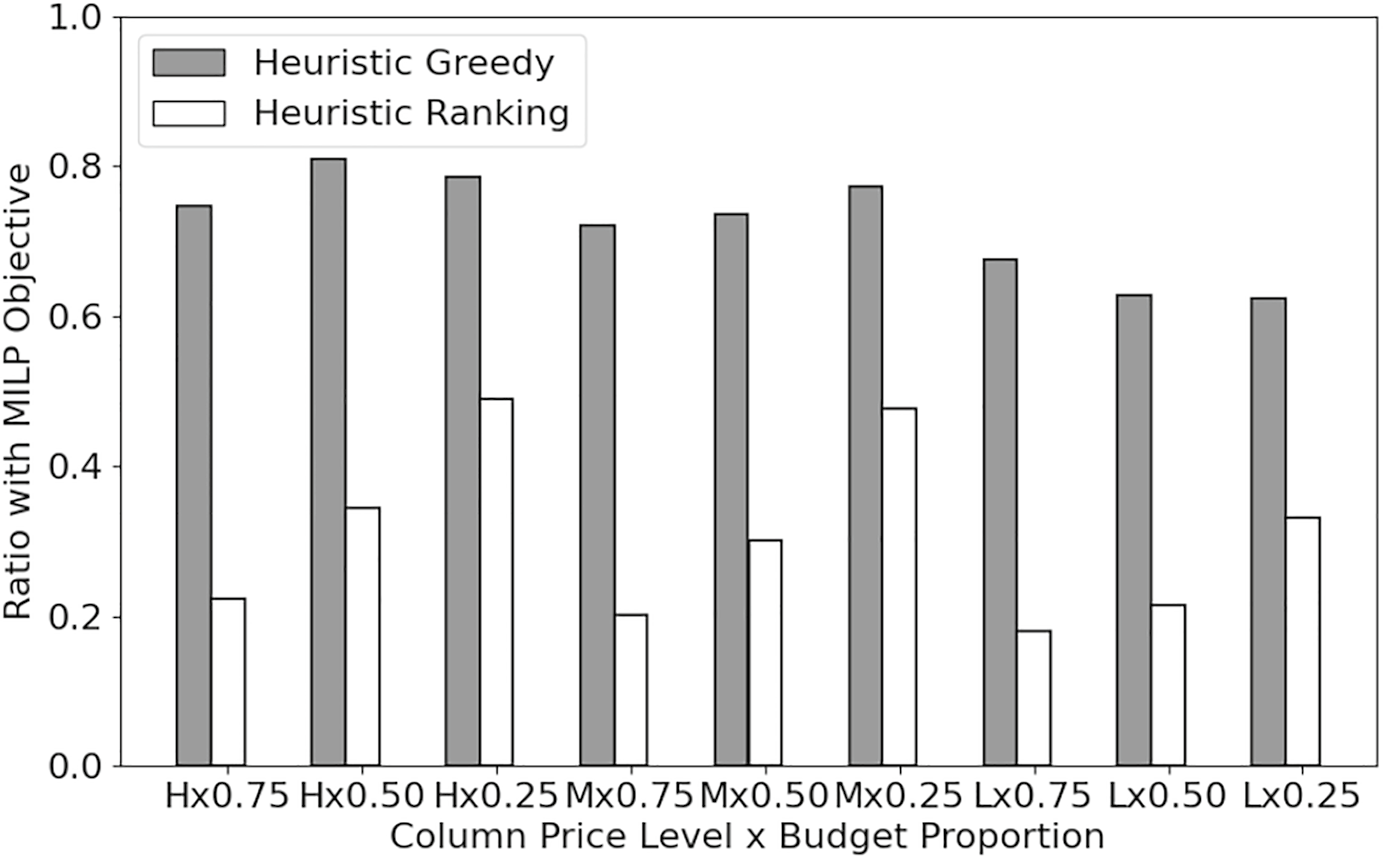
Quality of the heuristic solution methods

Details of our computational results for the two heuristic methods $H_\text {G}$ and $H_\text {R}$, in comparison with the MILP formulation (2), are provided in Appendix A. In summary, the MILP formulation () was most often faster than $H_\text {G}$. The $H_\text {G}$ solutions led to 20%-40% smaller game values than that of the MILP solutions, even for instances where MILP timed out. The $H_\text {R}$ method was much faster than MILP and $H_\text {G}$, as expected, but its relative gap (from an optimal solution) was almost twice as big as $H_\text {G}$. Figure 2 illustrates these results.

Lastly, we measured the strength of the conditional dominance inequality from Proposition 3. Including the inequality in the MILP formulation led to improved solution time for about 40% of the instances that were solved within the time limit. The maximum number of (branch-and-cut) nodes explored among the instances in each class was most often larger for the formulation without cuts, which implies the inequality may be more effective when employed in solving large-scale instances. Detailed computational results are presented in Appendix A.

## Conclusion

This paper introduces the matrix game designer problem (MGD), which finds applications in the security and advertising domains. We propose a mixed-integer linear programming formulation for MGD, present some analytical results, and provide results of our computational experiments.

## Data Availability

Data/code to replicate the computational experiments is available at https://github.com/raulgarcia66/Matrix-Game-Administrator

## Appendix A: Computational experiment results

Table 1 presents details of our computational experiment results for the heuristic methods, in comparison with the MILP formulation (2). Table 2 provides our computational results concerning the strength of the conditional dominance inequality of Proposition 3.

**Table 1:** Heuristic methods analysis

MILP	Column	Budget	Group	MILP Soln	$H_\text {G}$ Soln	$H_\text {R}$ Soln	$H_\text {G}$ Faster	$H_\text {R}$ Faster	$H_\text {G}$ Gap	$H_\text {R}$ Gap	MIPGap
status	prices	proportion	size	time	time (s)	time (s)	count	count	(%)	(%)	(%)
OPTIMAL	LOW	0.75	45	$< 1$	9.67	$< 1$	0	45	32.5	82.0	–
	LOW	0.50	45	46.62	9.19	$< 1$	13	45	37.3	78.6	–
	LOW	0.25	33	83.67	2.57	$< 1$	14	33	37.1	70.3	–
	MEDIUM	0.75	45	1.95	9.68	$< 1$	1	45	27.8	79.8	–
	MEDIUM	0.50	35	46.58	3.28	$< 1$	10	35	25.7	69.7	–
	MEDIUM	0.25	35	70.08	2.41	$< 1$	35	20	23.3	55.4	–
	HIGH	0.75	45	8.30	9.55	$< 1$	4	45	25.4	77.8	–
	HIGH	0.50	35	54.17	3.12	$< 1$	15	35	18.1	65.5	–
	HIGH	0.25	36	59.99	2.49	$< 1$	23	36	21.9	53.6	–
TIME_LIMIT	LOW	0.25	12	1200	17.48	$< 1$	12	12	38.9	60.1	39.5
	MEDIUM	0.50	10	1200	28.00	$< 1$	10	10	29.2	70.4	22.1
	MEDIUM	0.25	10	1200	19.76	$< 1$	10	10	20.6	42.0	32.1
	HIGH	0.50	10	1200	27.53	$< 1$	10	10	22.7	66.4	23.3
	HIGH	0.25	9	1200	20.62	$< 1$	9	9	19.4	41.4	16.6

We define the relative gap for each heuristic method *H*, denoted by *H* Gap (%), as the difference between its objective and the MILP objective, divided by the MILP objective. MIPGap is defined as the relative gap between the primal and dual bounds of the MILP solution, as returned by the solver. The “Faster” columns refer to being faster than the MILP formulation. We display the mean of these gaps for each group

**Table 2:** Conditional dominance inequality analysis

Status	Column	Budget	Group	Cuts Soln	No Cuts Soln	No Cuts	Cuts	Obj$_\text {c}>$ Obj$_\text {nc}$	N$_{\text {nc}}$ - N$_\text {c}$	N$_{\text {c}}$ - N$_{\text {nc}}$
(both)	prices	proportion	size	time (s)	time (s)	faster	faster	count	max	max
OPTIMAL	LOW	0.75	12	$< 1$	$< 1$	7	4	–	0	0
	LOW	0.50	12	16.27	16.06	5	7	–	326	83,997
	LOW	0.25	6	7.75	8.19	3	3	–	16,143	15,131
	MEDIUM	0.75	12	2.04	2.27	5	7	–	17,640	9,618
	MEDIUM	0.50	6	1.65	2.02	5	1	–	6,170	1,193
	MEDIUM	0.25	6	40.92	39.08	4	2	–	48,428	128,249
	HIGH	0.75	12	9.50	9.66	6	6	–	34,296	24,356
	HIGH	0.50	6	2.99	3.37	5	1	–	6,904	1,155
	HIGH	0.25	6	6.36	6.82	4	2	–	8,482	2,435
TIME_LIMIT	LOW	0.25	6	600	600	–	–	1	964,750	196,695
	MEDIUM	0.50	6	600	600	–	–	2	598,087	290,328
	MEDIUM	0.25	6	600	600	–	–	2	897,771	122,405
	HIGH	0.50	6	600	600	–	–	3	2,157,927	2,301,566
	HIGH	0.25	6	600	600	–	–	4	1,895,997	914,481

We excluded matrices with 10 rows or 10 columns, as solution times were small. With *N* denoting node count, the last two columns refer to the worse case additional nodes one method required over the other. We remark that the node count for the first group is 1 for all instances, as the problem was sufficiently easy to solve without a branch-and-cut procedure. We set the time limit of 10 min in these experiments

## Footnotes

1 A dominated strategy for a row player is a row of the matrix such that the payoff vector corresponding to that row is pointwise lower than the payoff vector corresponding to some other row.

2 It is impractical to have every passenger be screened by each type of security [10, 11].

3 Of course, more attractive strategies do not result in higher expected payoffs for the player, expectation taken over the optimal strategy of the opponent. It is straightforward to show that given the opponent’s optimal strategy, all actions in the support of a player’s optimal strategy must result in the same expected value, equaling the value of the matrix game. Hence we use the term attractiveness to simply denote actions that are played more often in an optimal strategy.
